# Short-Term Particulate Threat: Pollution Standard May Not Protect Health

**DOI:** 10.1289/ehp.115-a262b

**Published:** 2007-05

**Authors:** Bob Weinhold

Many studies have shown that particulate matter (PM) poses health risks, yet the attributes of PM that cause these effects remain uncertain. To address some of those critical nuances, especially the short-term effects of specific emissions, researchers used a refined approach, including new application of a pollutant distribution model, to assess links between deaths and two PM components, black carbon and sulfate particles **[*EHP* 115:751–755; Maynard et al.]**. They found that as the air concentration of either component increased, there were more deaths the following day. These results occurred even at concentrations below current U.S. standards for fine particulates.

Sulfate exposure in the northeastern United States comes in large part from coal-fired power plants. Black carbon is a surrogate for vehicle-related pollution that varies significantly over short distances. The researchers used data from a central monitor at the Harvard School of Public Health to determine concentrations of sulfates and assumed there were homogenous concentrations throughout the study area, a premise other studies have validated. To estimate concentrations of black carbon, they used a model that began its calculations with daily data from another monitor at the school. The model then estimated black carbon concentrations at more than 80 representative sites in the Boston area, incorporating variables such as weather, season, day of week, traffic volume, proximity to major roadways, population density, and percent urbanization. The researchers also accounted for gender, education, income, and residence location for each death.

In evaluating 107,925 deaths that occurred at Boston-area residences from 1995 through 2002, the researchers found that each interquartile increase in black carbon concentration on the day before death was linked with a 2.3% rise in deaths from any cause and a 4.4% increase in stroke deaths. A similar, though smaller, relationship existed for sulfate particles, with each interquartile increase the day before death linked with a 1.1% increase in death from any cause. The researchers also found that for both black carbon and sulfates, there were increases of similar magnitude for deaths from cardiovascular disease, respiratory diseases, and diabetes.

The authors acknowledge that the black carbon estimation model still needs refinement, that the study was limited by its focus on just one city, and that there were relatively limited data for sulfates and some causes of death. Nonetheless, this work confirms past research implicating sulfates and black carbon in the PM–mortality association. As a result, the authors say their findings reinforce concerns that current and proposed fine particulate standards do not adequately protect public health.

## Figures and Tables

**Figure:**
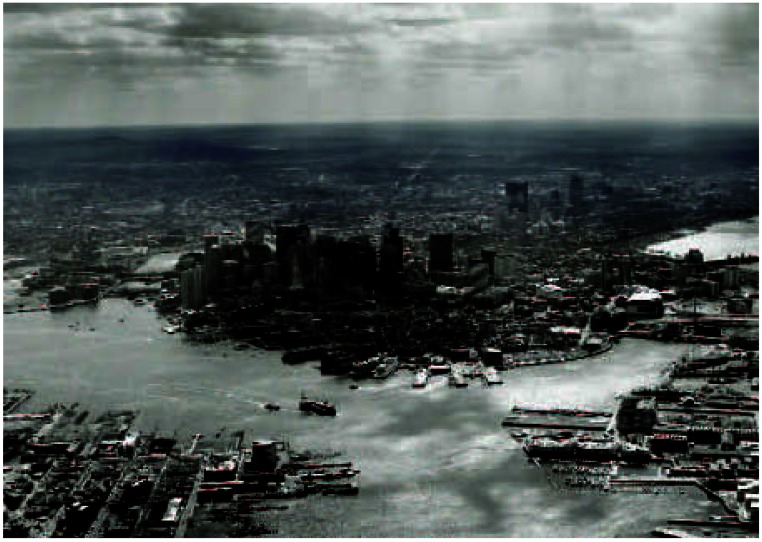
Boston, Massachusetts

